# Mechanism by Which PF-3758309, a Pan Isoform Inhibitor of p21-Activated Kinases, Blocks Reactivation of HIV-1 Latency

**DOI:** 10.3390/biom13010100

**Published:** 2023-01-04

**Authors:** Benni Vargas, James Boslett, Nathan Yates, Nicolas Sluis-Cremer

**Affiliations:** 1 Division of Infectious Diseases, Department of Medicine, University of Pittsburgh School of Medicine, Pittsburgh, PA 15261, USA; 2 Department of Cell Biology, University of Pittsburgh School of Medicine, Pittsburgh, PA 15213, USA; 3 Department of Chemistry, University of Pittsburgh School of Medicine; Pittsburgh, PA 15260, USA

**Keywords:** HIV-1, latency, p21-activated kinase, PF-3758309, NF-κB signaling

## Abstract

The “block and lock” strategy is one approach that might elicit a sterilizing cure for HIV-1 infection. The “block” refers to a compound’s ability to inhibit latent HIV-1 proviral transcription, while the “lock” refers to its capacity to induce permanent proviral silencing. We previously identified PF-3758309, a pan-isoform inhibitor of p21-activated kinases (PAKs), as a potent inhibitor of HIV-1 latency reversal. The goal of this study was to define the mechanism(s) involved. We found that both 24ST1NLESG cells (a cell line model of HIV-1 latency) and purified CD4+ naïve and central memory T cells express high levels of PAK2 and lower levels of PAK1 and PAK4. Knockdown of PAK1 or PAK2, but not PAK4, in 24ST1NLESG cells resulted in a modest, but statistically significant, decrease in the magnitude of HIV-1 latency reversal. Overexpression of PAK1 significantly increased the magnitude of latency reversal. A phospho-protein array analysis revealed that PF-3758309 down-regulates the NF-κB signaling pathway, which provides the most likely mechanism by which PF-3758309 inhibits latency reversal. Finally, we used cellular thermal shift assays combined with liquid chromatography and mass spectrometry to ascertain whether PF-3758309 off-target binding contributed to its activity. In 24ST1NLESG cells and in peripheral blood mononuclear cells, PF-3758309 bound to mitogen-activated protein kinase 1 and protein kinase A; however, knockdown of either of these kinases did not impact HIV-1 latency reversal. Collectively, our study suggests that PAK1 and PAK2 play a key role in the maintenance of HIV-1 latency.

## 1. Introduction

Despite effective antiretroviral therapy (ART), a reservoir of latent, replication-competent proviral HIV-1 DNA persists in resting CD4+ T cells and potentially other cell types such as myeloid cells, which represents a major barrier to either a sterilizing or functional cure for HIV-1 infection [1,2,3]. While a sterilizing cure involves the complete elimination of the latent replication-competent proviral HIV-1 DNA in the body, a functional cure results in the long-term control of HIV-1 replication in the absence of ART. The “block and lock” therapeutic strategy represents one approach for a functional cure. This strategy involves long-term silencing of HIV-1 proviruses to prevent viral rebound following therapy cessation [4,5]. The “block” refers to therapeutic agents, or latency promoting agents, that inhibit (or “block”) the transcription of latent HIV-1 proviruses. Latency promoting agents can specifically target viral proteins (i.e., Tat or integrase) and, in addition to their “blocking” phenotype, they may also elicit antiviral activity (i.e., inhibit ongoing HIV-1 replication). Alternatively, latency promoting agents could target host proteins and elicit only a “blocking” phenotype. The “lock” refers to the agent’s ability to induce permanent silencing of HIV-1 proviruses, typically via repressive epigenetic modifications [4,5].

Signaling pathways play a key role in HIV-1 latency [6]. In a recent study, our laboratory used the 24ST1NLESG cell line model of HIV-1 latency to screen a library of structurally diverse, medicinally active, cell permeable kinase inhibitors, which target a wide range of signaling pathways, to identify inhibitors of HIV-1 latency reversal [7]. The screen was carried out in the absence or presence of three mechanistically distinct latency-reversing agents, namely, prostratin, panobinostat, and JQ-1. We identified 12 kinase inhibitors that blocked the reversal of HIV-1 latency, irrespective of the latency reversal agent used in the screen. Of these, PF-3758309 was found to be the most potent. The 50% inhibitory concentrations in the 24ST1NLESG cells ranged from 0.1 to 1 nM (selectivity indices >3300). PF-3758309 was also found to inhibit latency reversal in CD4+ T cells isolated from HIV-1-infected donors on ART. The mechanism by which PF-3758309 inhibited reactivation of latent HIV-1 was not elucidated in this study.

**Figure 1:**
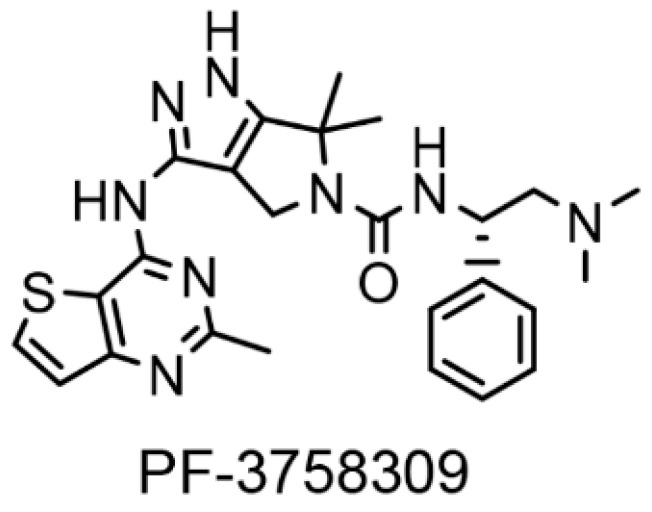
Chemical structure of PF-3758309.

PF-3758309 (Figure 1) is a potent, ATP-competitive, pyrrolo-pyrazole inhibitor of the p21-activated kinases (PAKs) with 50% inhibitory concentrations (IC_50_) of 13.7, 190, 99, 18.7, 18.1, and 17.1 nM against PAK 1, 2, 3, 4, 5, and 6 in cell-free assays, respectively [8]. PAKs are a family of serine/threonine-specific protein kinases that act as downstream effectors of the small GTPases Cdc42 and Rac, mediating an important subset of the signaling activities of these enzymes, and aberrant PAK signaling has long been associated with a number of human diseases [9]. Furthermore, there is emerging evidence that PAKs also play a major role in the entry, replication, and spread of many important pathogenic human viruses, including HIV-1 [10,11,12,13]. In regard to HIV-1, the viral accessory protein Nef specifically associates with PAK2, and this interaction appears to be critical for several aspects of HIV-1 biology, not only directly affecting virus replication but also indirectly promoting virus spread and persistence via disturbance of key players in the antiviral immune response [11,13]. The primary objective of this study was to determine how inhibition of the different PAK isoforms by PF-3758309 modulates HIV-1 latency.

## 2. Materials and Methods

### 2.1. Reagents

The 24ST1NLESG cell line of HIV-1 latency was kindly provided by Dr. Joseph P. Dougherty (Robert Wood Johnson Medical School). Analysis of HIV-1 latency reversal in the 24ST1NLESG cells was conducted as described previously [14]. Briefly, in the 24ST1NLESG cell line, the integrated HIV-1 genome encodes a secretable alkaline phosphatase (*SEAP*) gene in *env*, which serves as an indicator of late gene expression, and used a measure of latency reversal. PF-3758309 was purchased from Selleckchem (Houston, TX, USA). siRNA molecules were obtained from Dharmacon^TM^ (Horizon Discovery Ltd., Co., USA). All other reagents were of the highest quality available and were used without further purification.

### 2.2. Quantification of PAK Expression

RNA was extracted from purified CD4+ T_N_ and T_CM_ cells and from 24ST1NLESG cells using the RNeasy mini kit (Qiagen, Valencia, CA, USA) according to the manufacturer’s protocol. Transcriptome analysis for the CD4+ T_N_ and T_CM_ cells was carried out by RNA sequencing (RNA-Seq; GENEWIZ, Azenta Life Sciences, South Plainfield, NJ, USA). The RNA from 24ST1NLESG cells was DNase-treated with RNase-Free DNase (Qiagen, Valencia, CA, USA) and reverse transcribed using random primers with the AffinityScript multiple temperature reverse transcriptase (Agilent, Wilmington, DE, USA). Quantitative PCR (qPCR) was performed using a Bio-Rad CFX96 real-time PCR detection system, using 2× Lightcycler 480 probes master (Roche, Indianapolis, IN, USA) for IPO8 and Maxima SYBR Green/ROX qPCR Master Mix (2×) (Thermofisher, Waltham, MA, USA) for PAK, using primers described in Supplementary Table S1. Quantification for each qPCR reaction was assessed by the 2^−Δ*CT*^ algorithm, relative to IPO8 expression.

### 2.3. siRNA Transfection of 24ST1NLESG Cells

The 24ST1NLESG cells were transfected by electroporation using the Neon Transfection System (Life Technologies, Carlsbad, CA, USA). Electroporation parameters were 1350 Pulse Voltage (V), 10 Pulse Width (ms), 3 Pulse Number, 2 × 10^7^ cell/mL. Final concentrations were 1 × 10^7^ cell/mL, 1 µM siRNA (siPAK1, Dharmacon, cat # L-003521-00-0020; siPAK2, Dharmacon, cat # L-003597-00-0020; siPAK4, Dharmacon, cat # L-003615-00-0020; siMAPK1, Thermofisher cat # 4390824; siPKA, Thermofisher cat # 4390825; Non-targeting Control, Dharmacon, cat # D-001810-10-20). The efficiency of siRNA knockdown was assessed by Western blot analysis. Anti-PAK1 (cat # 2602S), anti-PAK2 (cat #2608S), anti-PAK4 (cat # 62690), anti-PKA (cat # 4782), anti-MAPK (cat # 9108), and anti-GAPDH (cat # 97166S) antibodies were purchased from Cell Signaling. The efficiency of siRNA knockdown was quantified using ImageJ.

### 2.4. PAK1 and PAK2 Overexpression

Transfection of 24ST1NLESG cells was carried out with 8 µg PAK1 expression plasmid (Addgene, Watertown, MA, USA, cat # 12208), PAK2 expression plasmid (Sino Biologicals, cat # HG10085-UT), or 8 µg empty vector (Sino Biologicals, cat # CV011). Cells were harvested 120 h post-transfection for Western blot to assess overexpression efficiency.

### 2.5. Phospho Explorer Antibody Array

An amount of 5 × 10^6^ 24ST1NLESG cells exposed to 8 nM PF-3758309 or DMSO (control) were sent to Full Moon Biosystems (Sunnyvale, CA, USA) for analysis. In our data analysis, Phospho-ratios are changes between PF-3758309-treated vs. DMSO control where the ratio is equal to the signal intensity of the Phospho Site-Specific Antibody over the signal intensity of the site-specific antibody. Phospho-ratios of an absolute fold change >1.4 or <0.6 were included in the analysis. *p*-values were calculated using the Fisher’s Exact Test. Ingenuity^®^ Pathway Analysis (IPA; QIAGEN, Redwood city, CA, USA) was used to further analyze the data. The software mapped each of the proteins to the repository of information in the Ingenuity Pathways Knowledge base. Molecular networks and canonical pathways regulated by PF-3758309 were obtained using IPA core analysis.

### 2.6. Cellular Thermal Shift Assays

Cell lysates from 24ST1NLESGs or PBMC were treated with DMSO (vehicle) or 10 µM PF-3758309 and subjected to cellular thermal shift assays, as described previously [15]. For each cell type, an individual CETSA experiment was performed at 50, 55, and 60 °C to cover the majority of the mammalian proteome melting curve [16]. The extracts were adjusted to 1 mg/mL protein concentrations and were divided into 50 μL aliquots (*n* = 6) of compound and vehicle-treated samples. The samples were heated in parallel at a fixed temperature for 10 min, followed by a 5-min incubation at room temperature. Samples were then centrifuged at 25,000× *g* for 10 min at room temperature, and the supernatant denatured and digested with trypsin utilizing the FASP method [17]. Samples were freeze-dried in a vacuum concentrator and resuspended in 30 μL 0.1% formic acid in water. Nanoflow LC-MS/MS analysis was performed on a Waters NanoAcquity system (Milford, MA, USA). Samples (1 μL) were injected via autosampler onto a 25 cm × 75 μM ID reversed phase column packed with 3 μM Reprosil (New Objective, Boston, MA, USA). Peptides were separated and eluted with a gradient from 2 to 32% acetonitrile in 0.1% formic acid over 70 min at 300 nL/min into an LTQ Orbitrap XL hybrid mass spectrometer (Thermo Fisher Scientific, Waltham, MA, USA) using a data-dependent top 8 method in positive ion mode, with spray voltage set at 2.0 kV. Full scan spectra were acquired in the range of *m*/*z* 350–1600 at 60,000 resolution using an automatic gain control target of 1 × 10^6^. Tandem mass spectra were acquired in the linear ion trap with a 35% normalized collision energy setting and an MS/MS ion target of 5 × 10^4^. Mass spectrometry data were analyzed using MaxQuant (version 1.6.7.0). Spectra were searched against the Uniprot reference database using the MaxQuant built-in peptide identification algorithm, Andromeda. Trypsin was specified as the digestion protease with the possibility of two missed cleavages. Acetylation (protein N-terminus) and oxidation of methionine were set as default variable modifications while carbamidomethylation of cysteine residues was set as a fixed modification. Other database search parameters included 20 ppm and 0.5 Da mass tolerances for precursor and product ions, respectively. Intensities for all peptides were assigned by MaxQuant using full scan mass spectra. Quantified peptides with large within replicate variability were filtered using a minimum occupancy filter (real values were required in a minimum of three out of six replicates for inclusion). The MaxQuant Peptide Groups file was then analyzed using Microsoft Excel, and statistical significance was established using the Student’s *t* test on peptide peak intensity values. The GraphPad Prism software was used to generate a volcano scatterplot of the statistical significance versus magnitude of change for each protein group at a given temperature. Proteins were considered as “hits” if the relative abundances of two peptides were found with *p*-values < 0.001 between vehicle and PF-3758309-treated groups.

## 3. Results

### 3.1. PAK Isoform Expression in 24ST1NLESG Cells and in Purified CD4+ Naïve (T_N_) and Central Memory (T_CM_) T Cells

**Figure 2:**
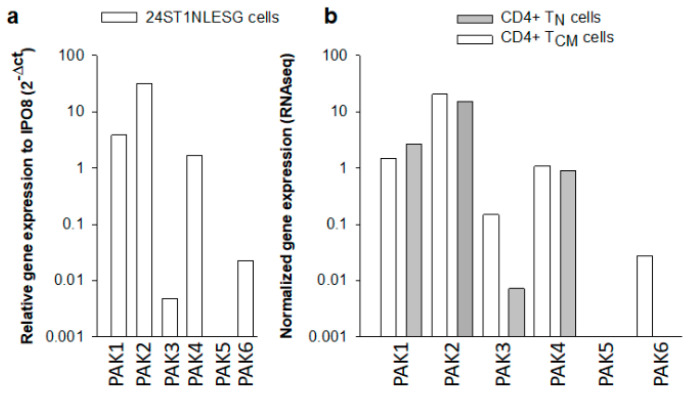
RNA expression levels of different PAK isoforms in 24ST1NLESG cells (**a**) and in purified naïve (T_N_) and central memory (T_CM_) CD4+ T cells. PAK RNA expression is reported relative to cellular IPO8 expression. Data in Figure 2a represent the mean from two independent experiments. Data in (**b**) are from a single RNAseq experiment.

PF-3758309 is an orally available, potent, ATP-competitive, pyrrolopyrazole inhibitor of the PAKs, which are involved in physiological processes including motility, survival, mitosis, transcription, and translation [9]. Depending on structural and functional similarities, the six members of the PAK family are divided into two groups, with three members in each group [9]. Group I PAKs (1–3) are activated by extracellular signals through GTPase-dependent and GTPase-independent mechanisms. In contrast, group II PAKs (4–6) are constitutively active. PF-3758309 exhibits inhibitory activity toward all of the PAK isoforms. We used quantitative PCR or population RNA sequencing (RNAseq) data to assess PAK isoform expression in 24ST1NLESG cells and purified CD4+ T_N_ and T_CM_ cells (Figure 2). Of note, CD4+ T_N_ and T_CM_ cells are regarded as major reservoirs of latent HIV-1 infection in infected individuals on ART [1,2]. In the 24ST1NLESG cell line, we noted robust expression of PAK2 and somewhat lower expression levels of PAK1 and PAK4 (Figure 2a). Expression of PAK3 and PAK6 was exceptionally low, and we did not detect expression of PAK5 (Figure 2a). The PAK isoform expression levels in CD4+ T_N_ and T_CM_ cells were similar to that of the 24ST1NLESG cell line (Figure 2b).

### 3.2. Knockdown of PAK1, PAK2, and PAK4 in 24ST1NLESG Cells

**Figure 3:**
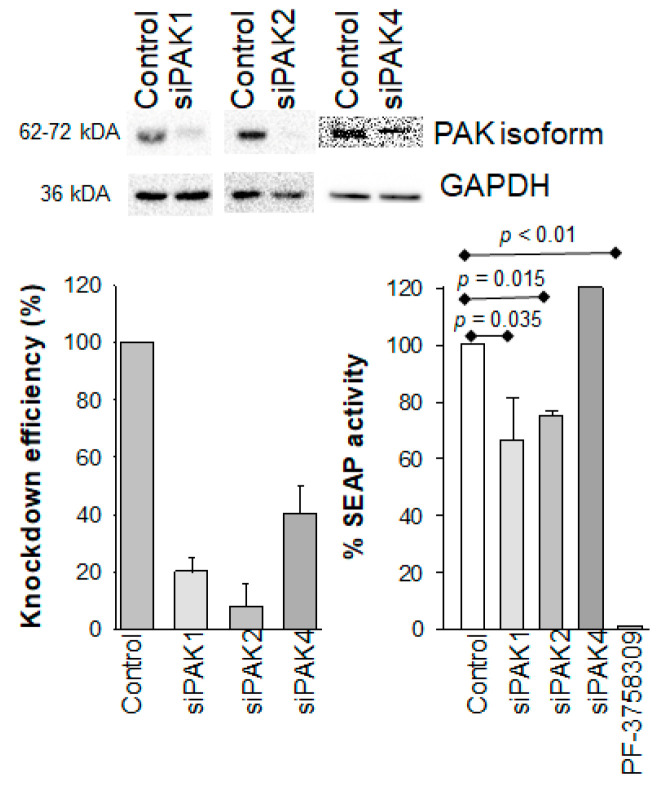
Effect of PAK1, PAK2, and PAK4 knockdown of HIV-1 latency reversal in 24ST1NLESG cells. (a) Representative Western blot analysis of PAK knockdown; (b) quantification of PAK knockdown by Western blot using ImageJ; (c) effect of PAK knockdown on HIV-1 latency reversal mediated by TNF-α in 24ST1NLESG cells. Data are shown as the mean ± standard deviation from at least three independent biological replicates. Statistical significance evaluated using Mann–Whitney U test.

We used siRNA to knock down expression of PAK1, PAK2, and PAK4 in 24ST1NLESG cells (Figure 3). A scrambled siRNA sequence was used as a negative control in these experiments. The magnitude of the siRNA-mediated gene silencing was confirmed 120 h post-siRNA transfection by Western blot analysis (Figure 3(a,b)). Knockdown of PAK1, PAK2, or PAK4 did not result in any measurable cellular cytotoxicity. Next, we assessed the effect of PAK isoform knockdown on the reversal of HIV-1 latency in 24ST1NLESG cells (72 h post-siRNA transfection) by addition of 50 ng/mL tumor necrosis factor α (TNFα) for 48 h (Figure 3(c)). Knockdown of PAK1 and PAK2 resulted in a modest, but significant, decrease in SEAP expression. Knockdown of PAK4 did not impact reactivation of latent HIV-1 expression. Combination knockdown studies (i.e., simultaneous knockdown of PAK1 and PAK2, PAK1 and PAK4, and PAK2 and PAK4) significantly decreased cellular viability, thus preventing detailed analyses of HIV-1 latency. These data provide evidence that inhibition of PAK1 and PAK2 contributes to the maintenance of latent HIV-1 infection in 24ST1NLESG cells.

### 3.3. Overexpression of PAK1 and PAK2 in 24ST1NLESG Cells

**Figure 4:**
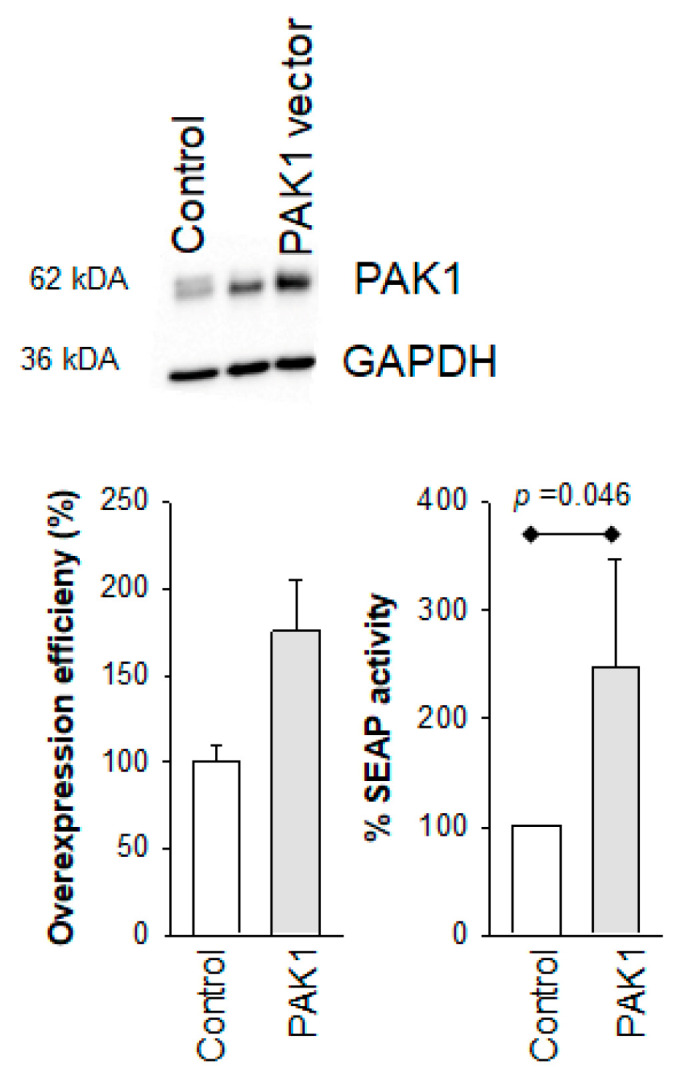
Effect of PAK1 overexpression on HIV-1 latency reversal in 24ST1NLESG cells. (a) Representative Western blot analysis of PAK1 overexpression; (b) quantification of PAK1 overexpression by Western blot using ImageJ; (**c**) effect of PAK1 overexpression on HIV-1 latency reversal mediated by TNF-α in 24ST1NLESG cells. Data are shown as the mean ± standard deviation from at least three independent biological replicates. Statistical significance evaluated using Mann–Whitney U test.

To further delineate the roles of PAK1 and PAK2 in the maintenance of HIV-1 latency, we overexpressed these isoforms in 24ST1NLESG cells (Figure 4). Cells were harvested 120 h post-transfection to assess overexpression by Western blot analysis. Compared to the control experiment, which included cells transformed with an empty vector, we observed notable overexpression of PAK1 in the in 24ST1NLESG cells (Figure 4(a,b)). By contrast, we could not assess PAK2 overexpression due to the experimental difficulty in purifying high plasmid yields in bacteria, as reported previously [18,19]. Overexpression of PAK1 in 24ST1NLESG cells resulted in a statistically significant increase in SEAP expression (Figure 4(c)), providing additional evidence that this isoform contributes to the maintenance of HIV-1 latency.

### 3.4. PAK Signaling and HIV-1 Latency Reversal

**Figure 5:**
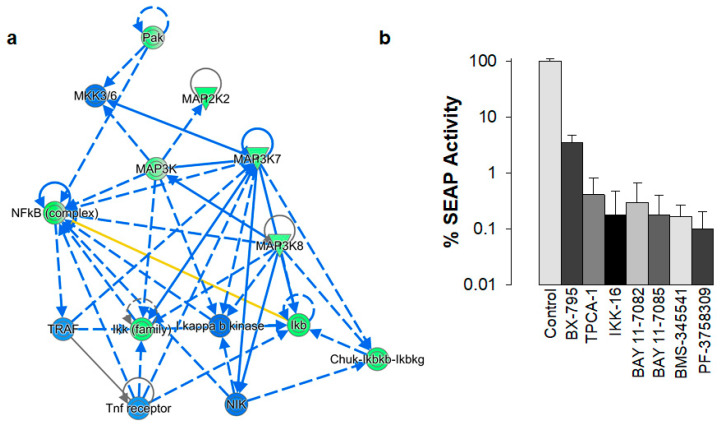
PF-3758309 and NF-κB signaling in 24ST1NLESG cells. (**a**) Qiagen Ingenuity Pathway Analysis was used to generate the functional network of the Phospho Antibody Array. The NF-κB signaling pathway was found to be significantly down-regulated (*p*-value 2.83 × 10^−24^). Lines and arrows between nodes represent direct (solid lines) and indirect (dashed lines) interactions between molecules as supported by information in the Ingenuity knowledge base. Green nodes represent decreased measurement in the dataset. Blue color represents predicted inhibition. The yellow line indicates findings inconsistent with state of downstream molecule. The gray line indicates effect not predicted. The circular line arrow indicates that a molecule interacts with itself. (**b**) Characterization of NF-κB inhibitors on HIV-1 latency mediated by TNF-α in the 24ST1NLESG cell line. Six different inhibitors (BX-795, TPCA-1, IKK-16, BAY11-7082, BAY11-7085, and BMS-345541) were used at a concentration of 2 µM. No cellular toxicity was observed. PF-3758309 was used as a concentration of 100 nM. Data are shown as mean ± standard deviation from at least three independent biological replicates.

PAKs act as key signal transducers in several signaling pathways, including Ras, Raf, NF-κB, Akt, Bad, and p53 [9]. Protein phosphorylation plays an important role in cell signaling. Accordingly, we used the Phospho Explorer Antibody Array (Full Moon Biosystems) to quantitate broad-scope phosphorylation profiling changes in 24ST1NLESG cells exposed to 8 nM PF-3758309 or DMSO (control) for 24 h to try to better understand how inhibition of PAKs by PF-3758309 controls HIV-1 latency. The Phospho Explorer Antibody Array conducts phosphorylation profiling with 1318 site-specific antibodies from over 30 signaling pathways. In our data analysis plan, we only considered phosphorylation changes that exhibited fold changes in phosphorylation that were >1.4 and <0.6 between control and PF-3758309 treated cells. In total, we identified 47 proteins that exhibited site-specific increased phosphorylation and 14 proteins that exhibited site-specific decreased phosphorylation in PF-3758309 treated cells, respectively (Table 1). Next, we used the Ingenuity^®^ Pathway Analysis to identify which signaling pathways were regulated by PF-3758309 (Figure 5a). This analysis revealed down-regulation of the NF-κB pathway (*p*-value 2.8 × 10^−24^), which is consistent with prior studies demonstrating that PF-3758309 suppressed NF-κB signaling in lung cancer cells [8,20]. Importantly, multiple studies have demonstrated that both the canonical and non-canonical NF-κB signaling pathways drive HIV-1 proviral expression [21]. To further evaluate the role of NF-κB signaling in the reversal of HIV-1 latency mediated by TNF-α in 24ST1NLESG cells, we treated cells with 2 µM of 6 different NF-κB inhibitors and then assessed SEAP activity (Figure 5b). None of the inhibitors elicited cellular cytotoxicity at the concentration used. All of the NF-κB inhibitors blocked reactivation of latent HIV-1 to the same extent as PF-3758309. As such, down-regulation of the NF-κB pathway by PF-3758309 provides the most likely mechanism by which the drug inhibits latency reversal.

### 3.5. Potential Off-Target Effects of PF-3758309

**Figure 6:**
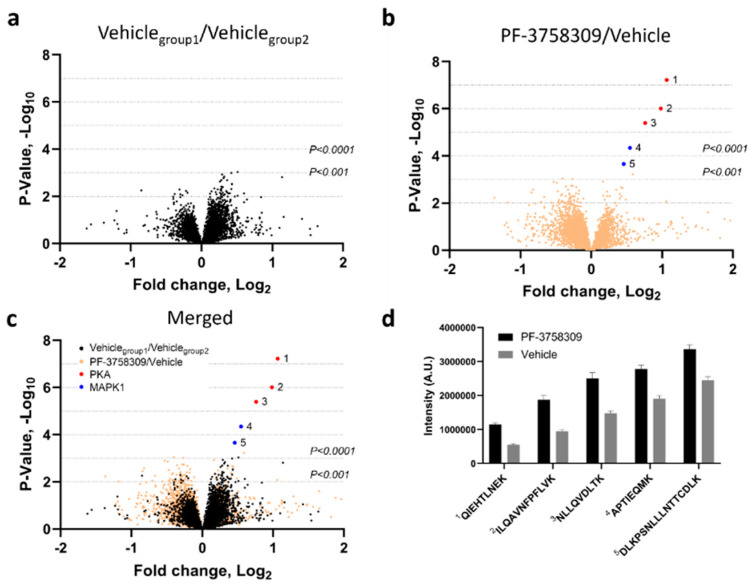
Drug/protein binding analysis of PF-3758309-treated 24ST1NLESG cells. Volcano plots for (**a**) two identical vehicle groups (decoy comparison), (**b**) PF-3758309 and vehicle groups (experimental comparison), and (**c**) the decoy and experimental comparisons merged. The x-axis indicates the relative fold change (log_2_-transformed expression values of peptides in the absence or presence of PF-3758309) and the y-axis indicates the –log10 of the *p* values. (**d**) Bar graph illustrating enrichment of PKA and MAPK1 peptides in 24ST1NLESG cell lysates treated with PF-3758309. Bar graph data are shown as the mean ± standard error.

Kinase inhibitors, such as PF-3758309, can produce off-target effects by inducing changes in molecules other than the one specifically targeted. Such off-target effects are generally attributed to non-specific binding or to cross-talk. PF-3758309 is an exceptionally potent inhibitor of HIV-1 latency reversal, with an IC_50_ for inhibition of latency reversal ranging from 0.070 to 1 nM depending on the latency reversing agent used (Table 1). As described above, knockdown or overexpression of PAK1 or PAK2 only provided a modest effect on latency reversal, possibly suggesting that the drug may bind to and inhibit other proteins also involved in the maintenance of HIV-1 latency. To identify possible cellular off-targets for PF-3758309, we used cellular thermal shift assays (CETSA) combined with liquid chromatography and mass spectrometry (LC-MS/MS). CETSA relies on the principle that small molecule binding to a protein alters the melting behavior of that protein, typically increasing it [22]. Briefly, CETSA involves treating cellular protein extracts with a test drug or its vehicle followed by heating to induce protein denaturation. Protein aggregates are pelleted by centrifugation, and the protein in the supernatant is proteolytically digested to peptides which are quantified by LC-MS/MS analysis. Peptides enriched in the drug-treated extracts represent potential binding partners for the drug of interest, as these were stabilized by drug treatment. We prepared 1 mg/mL cytosolic extracts of 24ST1NLESG cells or peripheral blood mononuclear cells (PBMC), added vehicle (i.e., DMSO) or 10 µM PF-3758309, and performed CETSA at 3 temperatures (50, 55, and 60 °C). LC-MS/MS was performed on six replicates from each of the conditions noted above. In some experiments, a decoy vehicle group was analyzed (vehicle vs. vehicle) to determine the statistical significance threshold for real “hits” in the experimental group (PF-3758309 vs. vehicle). Figure 6 and Supplementary Figure S1 show series of volcano plots for the 55 °C data in 24ST1NLESG cells (Figure 6) and peripheral blood mononuclear cells (Supplementary Figure S1), respectively. The vehicle vs. vehicle decoy comparison (panels 1a and 2a) shows similar thresholds required for statistical significance (~*p* < 0.001). From this, we established a practical filter for determining robust thermal stabilization events with PF-3758309 in the drug vs. vehicle comparison (panels 1b and 2b). This practical filter specified that, in order to be considered a robust thermal stabilization, the protein must have at least two peptides with levels statistically different (*p* < 0.001) between the PF-3758309 and vehicle-treated samples, as there were no proteins meeting this requirement in the vehicle vs. vehicle decoy group. Using this filter, we found two off-target binding proteins of PF-3758309, mitogen-activated protein kinase 1 (MAPK1) and protein kinase A (PKA). Interestingly, this finding was common to both cell types, as well as the analysis at 60 °C. Thus, the finding repeated across several independent experiments with varying proteomes and at different protein denaturation temperatures.

**Figure 7:**
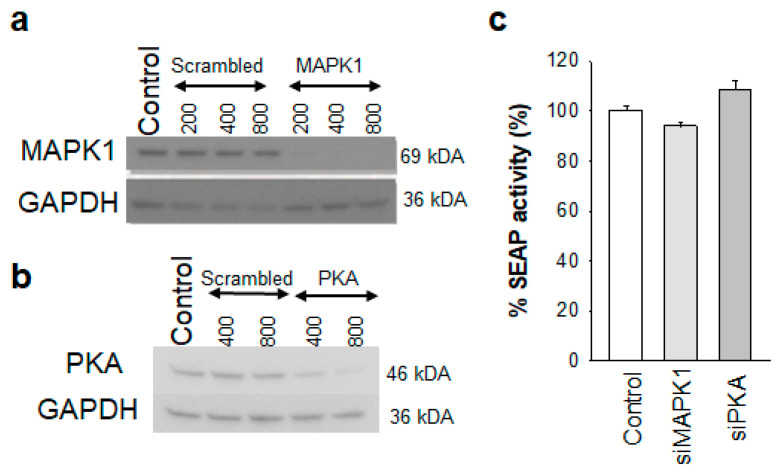
Effect of MAPK1 and PKA knockdown of HIV-1 latency reversal in 24ST1NLESG cells. (**a**) Western blot analysis of MAPK1 knockdown using different siRNA concentrations; (**b**) Western blot analysis of PKA knockdown using different siRNA concentrations; (**c**) effect of MAPK1 and PKA knockdown on HIV-1 latency reversal mediated by TNF-α in 24ST1NLESG cells. Data are shown as the mean ± standard deviation from at least three independent biological replicates. Statistical significance evaluated using Mann–Whitney U test.

Next, we used siRNA to knock down expression of MAPK1 and PKA in 24ST1NLESG cells (Figure 7). The magnitude of the siRNA-mediated gene silencing was confirmed 120 h post-siRNA transfection by Western blot analysis (Figure 7a,b). However, knockdown of MAPK1 and PKA did not impact HIV-1 latency reversal in 24ST1NLESG cells (Figure 7c), suggesting that off-target binding of PF-3758309 to these proteins does not contribute to the activity of the drug.

## 4. Discussion

The persistence of latent, replication-competent HIV-1 proviruses in resting CD4+ T cells represents a major barrier to curing HIV-1 infection [1,2]. To date, efforts to eradicate this viral reservoir via the shock and kill approach have not led to complete, long-term viral suppression in either cell or animal models. Thus, we need to consider alternate approaches that could lead to a sterilizing or functional cure for HIV-1 infection. The block and lock approach seeks to silence the transcriptional activity of latent proviruses, such that when ART is removed, viral rebound is significantly delayed or, better yet, prevented [4,5]. Several research groups have identified small molecule inhibitors that target different factors of the HIV-1 transcription machinery, leading to a block and lock phenotype [23]. However, blocking only one transcription pathway may not be sufficient to silence all proviruses, and thus it is likely that successful implementation of this strategy will require a combination of inhibitors. In this regard, there is a critical need to identify new molecules with different mechanisms of action. Our laboratory recently discovered that the pan isoform PAK inhibitor PF-3758309 is an exceptionally potent inhibitor of HIV-1 latency reactivation (IC_50_ in the pM to low nM range) with a huge selectivity index (>3000) [7]. In this study, we sought to define the role of different PAK isoforms in the maintenance and reversal of HIV-1 latency and to elucidate how PF-3758309 blocks HIV-1 latency reversal.

Our data show that CD4+ T cells express high levels of PAK2 and lower levels of PAK1 and PAK4. Knockdown of PAK1 or PAK2, but not PAK4, in 24ST1NLESG cells resulted in a modest, but statistically significant, decrease in the magnitude of HIV-1 latency reversal. Overexpression of PAK1 significantly increased the magnitude of latency reversal. The modest changes in HIV-1 latency reversal that were observed upon knockdown or overexpression of these PAK isoforms could be due to biological (i.e., their role in the maintenance and reversal of HIV-1 latency) and/or experimental parameters. In regard to the latter, the addition of TNF-α to reverse HIV-1 latency in 24ST1NLESG cells induces T cell proliferation, and as such, knockdown or overexpression of the PAK isoforms may be rapidly lost after addition of the cytokine. Interestingly, siRNA-mediated knockdown of PAK1 was previously shown to inhibit LTR-dependent transcription in human T-cell leukemia virus type 1 (HTLV-1) transformed MT4 cells and in cells transfected with an infectious clone of HTLV-1 [12]. The effect size observed in that study was similar to what we report here. Furthermore, a prior study also revealed that PAK1 depletion strongly inhibited HIV-1 infection in multiple cell systems and decreased levels of integrated provirus, and that overexpression of a constitutively active PAK1 enhanced HIV-1 infection [11]. Collectively, these studies highlight an important role for PAK1 and PAK2 in the maintenance of HIV-1 (and HTLV-1) latency.

PAKs act as key signal transducers in several signaling pathways, and protein phosphorylation plays an important role in cell signaling. We used the Phospho Explorer Antibody Array to quantitate broad-scope phosphorylation profiling changes in 24ST1NLESG cells exposed to PF-3758309. This analysis revealed that the inhibitor down-regulates the NF-κB signaling pathway, a finding that is consistent with prior published data [8,19]. NF-κB signaling has both antagonistic and agonistic effects on HIV-1 latency reversal [24]. Indeed, many HIV-1 latency reversing agents, including PKC agonists and SMAC mimetics, function by stimulating the NF-κB pathway (i.e., they act as agonists). In contrast, suppression or antagonism of the NF-κB pathway drives HIV-1 into latency. In our dataset, we see decreased phosphorylation of NF-κB p65 at residue T254 (Table 1). Phosphorylation of p65 at T254 stabilizes and promotes nuclear translocation of NF-κB [25]. We also observed decreased phosphorylation of IKKα/β at S180/S181 and NF-κB p100/p52 at S865. Phosphorylation of IKKα/β at S180/181is associated with increased RelA nuclear translocation, acetylation, DNA binding, and transactivation activity [26], while phosphorylation of NF-κB p100/p52 at S865 is also associated with activation of the NF- κB pathway [27]. Notably, six different NF-κB inhibitors all blocked reactivation of latent HIV-1 to the same extent as in PF-3758309 in 24ST1NLESG cells. Collectively, these data suggest that the primary mechanism by which PF-3758309 inhibits reversal of HIV-1 latency is via suppression of the NF-κB signaling pathway. Given that small molecule inhibitors can produce off-target effects via non-specific binding or cross-talk, we used CETSA LC-MS/MS to identify possible cellular off-targets of PF-3758309. While this analysis revealed that PF-3758309 was bound to mitogen-activated protein kinase 1 and protein kinase A, knockdown of either of these kinases did not impact HIV-1 latency reversal.

## 5. Conclusions

Taken together, our data show that PAK1 and PAK2 play a role in the maintenance of HIV-1 latency and further suggest that PAK inhibitors, such as PF-3758309, could form part of “block and lock” therapeutic strategies. In this regard, PF-3758309 was administered to patients with advanced solid tumors in a phase I clinical trial (ClinicalTrials.gov Identifier: NCT00932126). While the drug failed to advance due to the lack of an observed dose–response relationship, there were no safety concerns that contributed to the study termination. This suggests that PF-3758309 could be re-purposed for other clinical indications, including HIV-1.

## Figures and Tables

**Table 1:** Phospho-ratios between PF-3758309-treated and DMSO-treated 24ST1NLESG cells determined from Phospho Explorer Antibody Array data. The ratio is equal to the signal intensity of the Phospho site-specific antibody over the signal intensity of the site-specific antibody. Phospho-ratios of an absolute fold change > 1.4 or <0.4 were included in the analysis.

Protein (Phosphorylation Site)	Phospho Ratio	Protein (Phosphorylation Site)	Phospho Ratio
PKC zeta (Phospho-Thr560)	1.4	c-PLA2 (Phospho-Ser505)	1.62
Chk1 (Phospho-Ser345)	1.4	HSL (Phospho-Ser554)	1.64
EGFR (Phospho-Thr693)	1.41	FGFR1 (Phospho-Tyr766)	1.64
Paxillin (Phospho-Tyr31)	1.42	Raf1 (Phospho-Ser259)	1.66
Ephrin B1 (Phospho-Tyr317)	1.42	Cyclin D3 (Phospho-Thr283)	1.67
p53 (Phospho-Ser37)	1.42	IL3RB (Phospho-Tyr593)	1.68
PPAR-BP (Phospho-Thr1457)	1.43	Cyclin E1 (Phospho-Thr77)	1.68
BIM (Phospho-Ser69/65)	1.43	Stathmin 1 (Phospho-Ser15)	1.7
MEK1 (Phospho-Ser221)	1.44	Smad2/3 (Phospho-Thr8)	1.71
Chk2 (Phospho-Thr383)	1.45	Keratin 18 (Phospho-Ser52)	1.88
BAD (Phospho-Ser134)	1.45	B-RAF (Phospho-Thr598)	1.96
PKC theta (Phospho-Ser676)	1.46	Ephrin B2 (Phospho-Tyr330)	1.98
MEK1 (Phospho-Ser298)	1.47	c-Jun (Phospho-Thr93)	2.18
Calsenilin/KCNIP3 (Phospho-Ser63)	1.48	CD4 (Phospho-Ser433)	2.25
PKC alpha (Phospho-Tyr657)	1.48	HDAC1 (Phospho-Ser421)	2.38
HDAC2 (Phospho-Ser394)	1.48	p53 (Phospho-Ser315)	2.72
Synuclein alpha (Phospho-Tyr125)	1.49	Ras-GRF1 (Phospho-Ser916)	0.35
Estrogen Receptor-alpha (Phospho-Ser104)	1.49	Claudin 3 (Phospho-Tyr219)	0.43
IRS-1 (Phospho-Ser636)	1.49	BCR (Phospho-Tyr177)	0.44
Lamin A (Phospho-Ser22)	1.5	NFkB-p65 (Phospho-Thr254)	0.48
Cyclin D1 (Phospho-Thr286)	1.5	Caspase 9 (Phospho-Tyr153)	0.5
ALK (Phospho-Tyr1604)	1.52	Filamin A (Phospho-Ser2152)	0.51
Smad2 (Phospho-Ser467)	1.53	BID (Phospho-Ser78)	0.52
GSK3 alpha/beta (Phospho-Tyr216/279)	1.53	4E-BP1 (Phospho-Ser65)	0.52
14-3-3 zeta/delta (Phospho-Thr232)	1.53	PLCG1 (Phospho-Tyr771)	0.56
ERK3 (Phospho-Ser189)	1.53	ALK (Phospho-Tyr1507)	0.56
JunB (Phospho-Ser259)	1.54	HDAC3 (Phospho-Ser424)	0.56
c-Jun (Phospho-Tyr170)	1.58	Kv1.3/KCNA3 (Phospho-Tyr135)	0.57
LKB1 (Phospho-Ser428)	1.6	IKK-alpha/beta (Phospho-Ser180/181)	0.58
PP1 alpha (Phospho-Thr320)	1.61	NFkB-p100/p52 (Phospho-Ser865)	0.59
EGFR (Phospho-Tyr1069)	1.61		

## Data Availability

All data are available upon request.

## Supplementary Materials

The following supporting information can be downloaded at: https://www.mdpi.com/article/10.3390/biom13010100/s1, Figure S1: Drug/protein binding analysis of PF-3758309-treated peripheral blood mononuclear cells; Figure S2: Original Western blot data; Table S1: Primer sequences used for qPCR analysis of PAK isoform expression.
