# Supplementary material for: Mechanism by Which PF-3758309, a Pan Isoform Inhibitor of p21-Activated Kinases, Blocks Reactivation of HIV-1 Latency
Source: Biomolecules. 2023 Jan 4;13(1):100. doi: 10.3390/biom13010100 (PMC9855626; doi:10.3390/biom13010100)
Supplement: Supplementary file 1 [file biomolecules-13-00100-s001.zip › biomolecules-1954290-supplementary.pdf]

SUPPLEMENTARY DATA

Mechanism by which PF-3758309, a Pan Isoform Inhibitor of p21-Activated Kinases, Blocks Reactivation of HIV-1 Latency

Benni Vargas[a], James Boslett[b], Nathan A. Yates[b, c], Nicolas Sluis-Cremer[a]

[a] Division of Infectious Diseases, Department of Medicine, University of Pittsburgh School of Medicine, Pittsburgh, PA, 15261

[b] Department of Cell Biology, University of Pittsburgh School of Medicine, Pittsburgh, PA, 15261

[c] Department of Chemistry, University of Pittsburgh School of Medicine, Pittsburgh, PA, 15261

Supplementary Table 1: Primer sequences used for qPCR analysis of PAK isoform expression

Primer	Primer Sequence (5'→ 3')
PAK1 Fw	TATGATTGGAGCCGGCAGC
PAK1 Rv	CTGAAGGGAGAGAAATCTCTGGC
PAK2 Fw	CATCTTTAGCACTGGAGGCAAAGA
PAK2 Rv	ATGGTGTGCTCAAAATCAGATGG
PAK3 Fw	CAACCGGGATTCTTCAGCACT
PAK3 Rv	CACATGAATCGTATGCTCAAAGTCTG
PAK4 Fw	ATGTGGTGGAGATGTACAACAGCTA
PAK4 Rv	GTTCATCCTGGTGTGGGTGAC
PAK5 Fw	GGCGTCCTCTTGTGTCTTC
PAK5 Rv	GTACTGAGTCCTTCTGATTTGC
PAK6 Fw	CCTAGCCCTAAGACCCGGG
PAK6 Rv	GCGGTCGGAAAGAGGAGTTG
IPO8 Fw	GCTCTGATAACTGTGCAG
IPO8 Rv	CAGTGTGTACACCTCCTG
IPO8 Probe	FAM-TGCTGTCCTCTGATCCTCGC-TAMRA

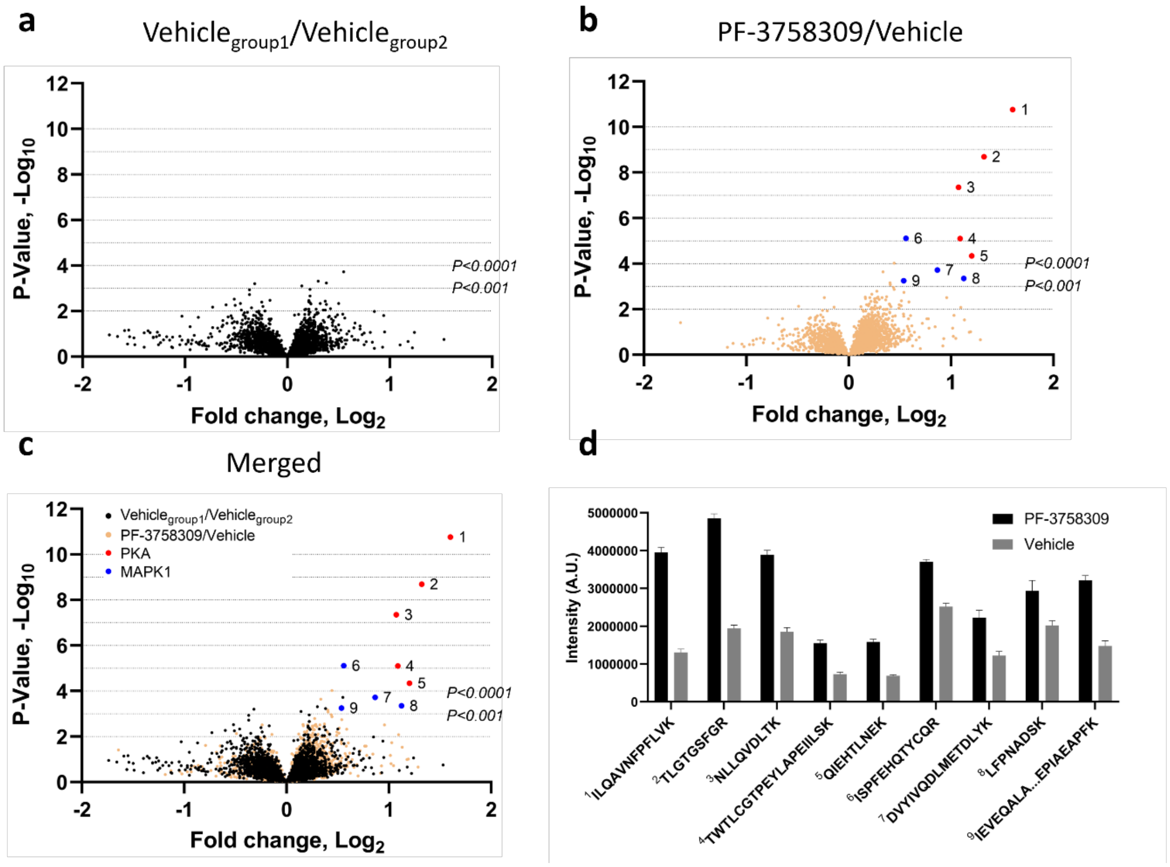

Supplementary Figure 1: Drug/protein binding analysis of PF-3758309-treated peripheral blood mononuclear cells. Volcano plots for (a) two identical vehicle groups (decoy comparison), (b) PF-3758309 and vehicle groups (experimental comparison), and (c) the decoy and experimental comparisons merged. The x-axis indicates the relative fold change ($\log_2$-transformed expression values of peptides in the absence or presence of PF-3758309) and the y-axis indicates the -log10 of the p values. (**d**) Bar graph illustrating enrichment of PKA and MAPK1 peptides in PBMC lysates treated with PF-3758309. Bar graph data are shown as the mean ± standard error.

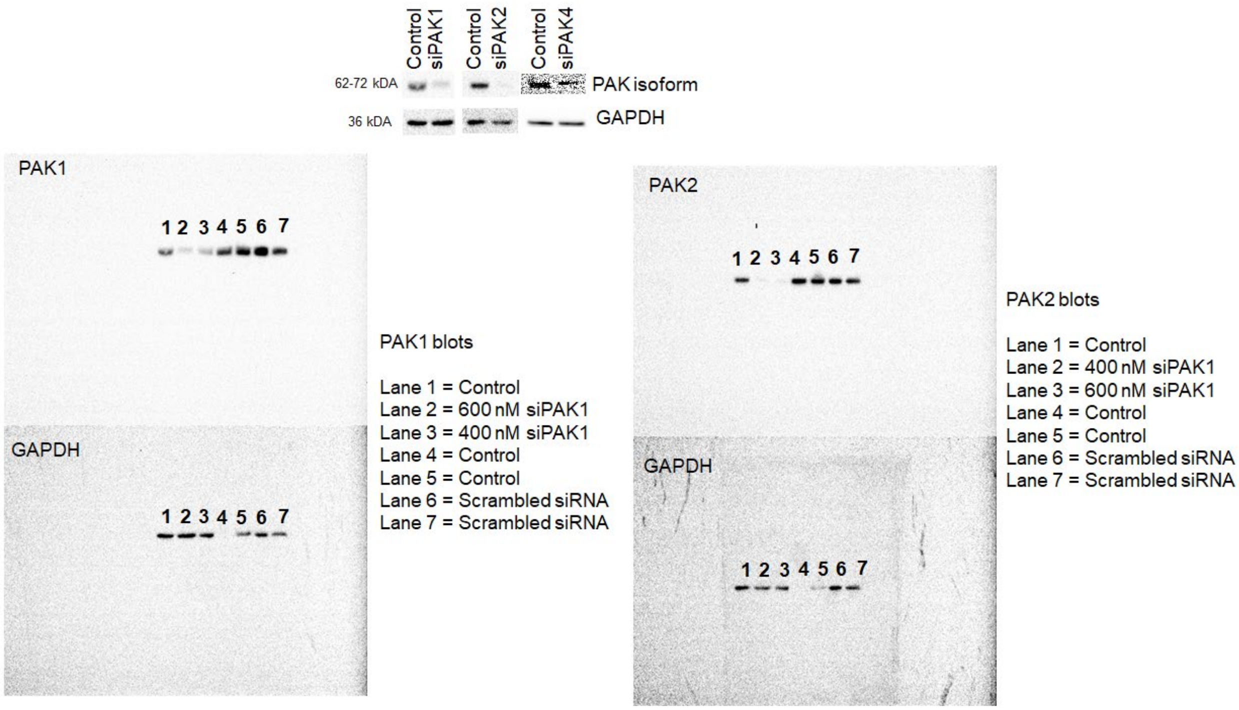

Supplementary Figure 2: Original Western Blot for knockdown of PAK1 and PAK2 in 24ST1NLESG cells.

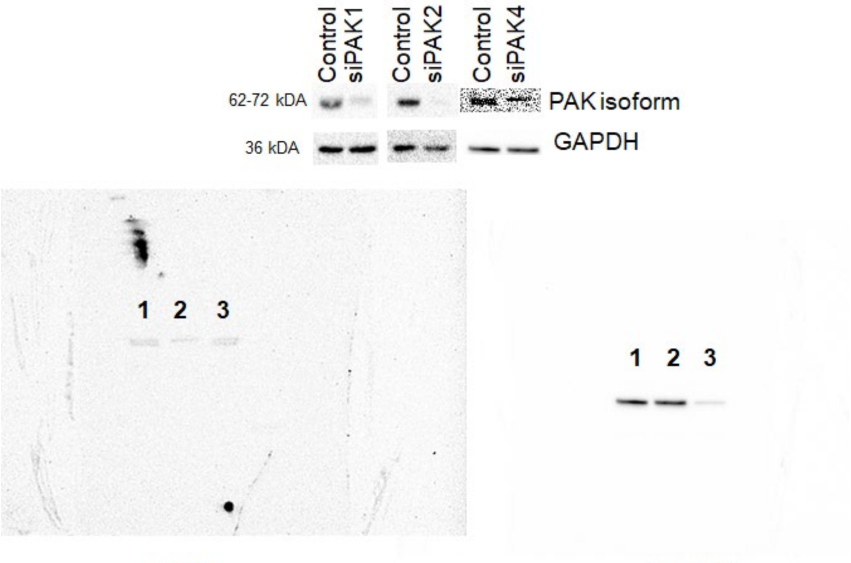

PAK4

Lane 1 = Control
Lane 2 = 600 nM siPAK4
Lane 3 = Control

GAPDH

Lane 1 = Control
Lane 2 = siPAK4
Lane 3 = Control

Supplementary Figure 3: Original Western Blot for knockdown of PAK4 in 24ST1NLESG cells.

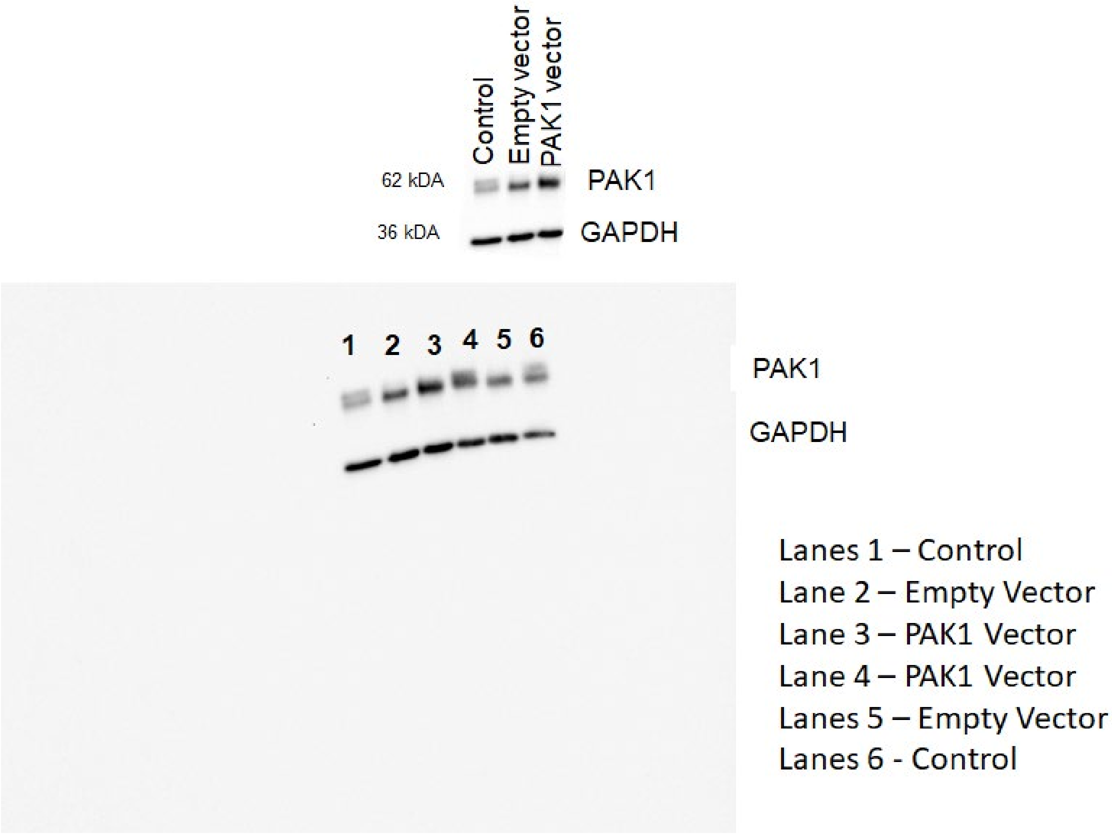

Lanes 1 – Control
Lane 2 – Empty Vector
Lane 3 – PAK1 Vector
Lane 4 – PAK1 Vector
Lanes 5 – Empty Vector
Lanes 6 - Control

Supplementary Figure 4: Original Western Blot for overexpression of PAK1 in 24ST1NLESG cells.

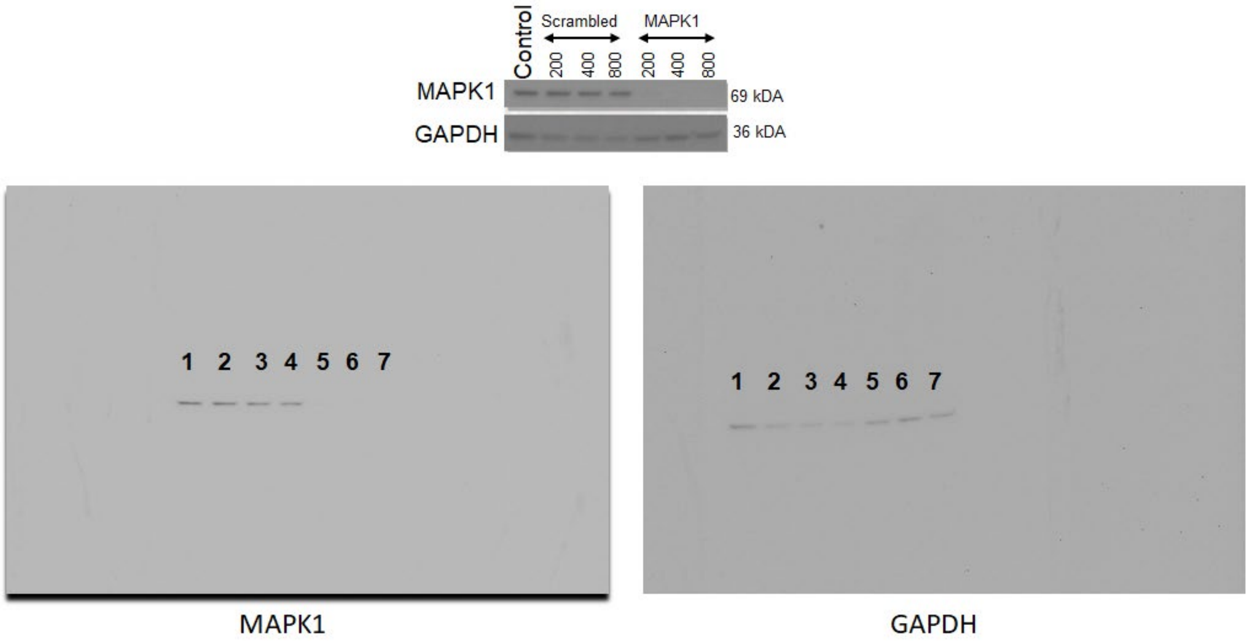

Supplementary Figure 5: Original Western Blot for knockdown of MAPK1 in 24ST1NLESG cells.

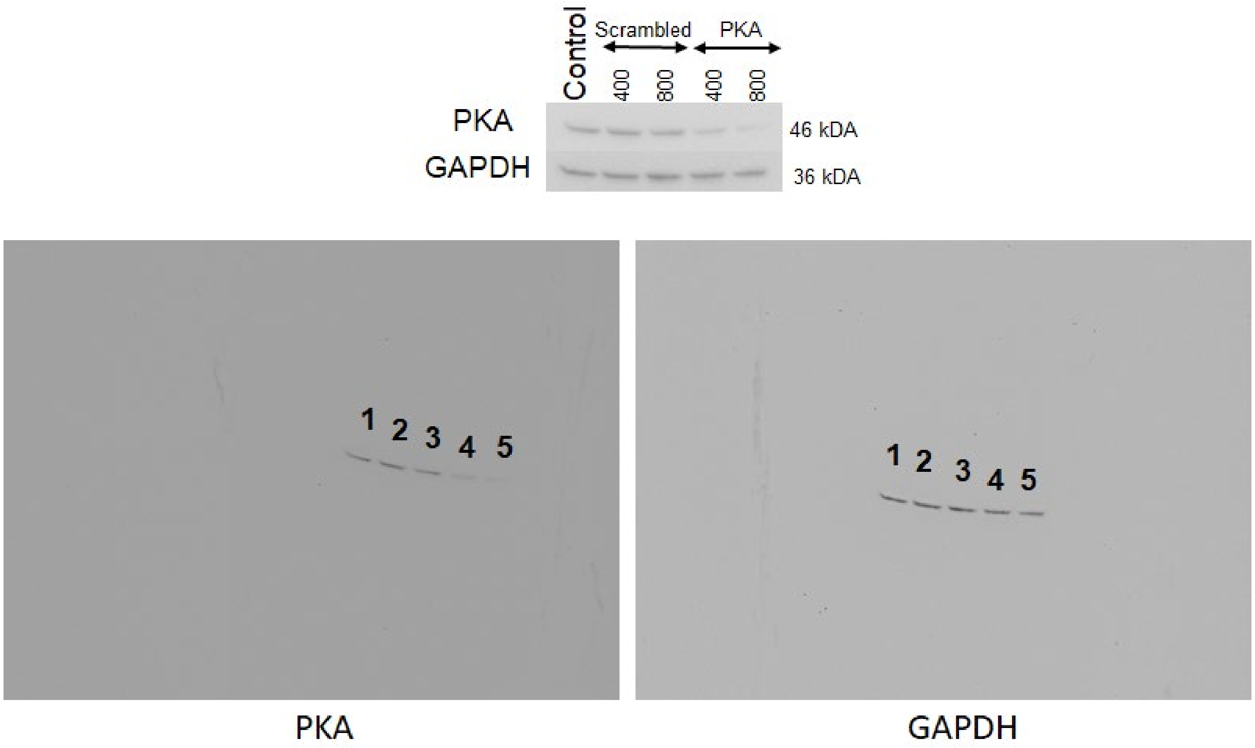

Supplementary Figure 2: Original Western Blot for knockdown of PKA in 24ST1NLESG cells.
